# The epidemiology of imported and locally acquired dengue in Australia, 2012–2022

**DOI:** 10.1093/jtm/taae014

**Published:** 2024-01-18

**Authors:** Asma Sohail, Katherine L Anders, Sarah L McGuinness, Karin Leder

**Affiliations:** School of Public Health and Preventive Medicine, Monash University, 553 St Kilda Road, Melbourne, Victoria 3004, Australia; Infectious Diseases Department, Grampians Health Service, 1 Drummond Street North, Ballarat, Victoria 3350, Australia; School of Public Health and Preventive Medicine, Monash University, 553 St Kilda Road, Melbourne, Victoria 3004, Australia; World Mosquito Program, Monash University, 12 Innovation Walk, Clayton, Victoria 3800, Australia; School of Public Health and Preventive Medicine, Monash University, 553 St Kilda Road, Melbourne, Victoria 3004, Australia; Infectious Diseases Department, Alfred Health, 55 Commercial Road, Melbourne, Victoria 3004, Australia; School of Public Health and Preventive Medicine, Monash University, 553 St Kilda Road, Melbourne, Victoria 3004, Australia; Victorian Infectious Diseases Service, Melbourne Health, 300 Grattan Street, Parkville, Victoria 3050, Australia

**Keywords:** Infectious disease, notifiable disease, arbovirus, surveillance, imported infection, travel

## Abstract

**Background:**

Dengue is the most important arboviral disease globally and poses ongoing challenges for control including in non-endemic countries with competent mosquito vectors at risk of local transmission through imported cases. We examined recent epidemiological trends in imported and locally acquired dengue in Australia, where the *Wolbachia* mosquito population replacement method was implemented throughout dengue-prone areas of northern Queensland between 2011 and 2019.

**Methods:**

We analysed dengue cases reported to the Australian National Notifiable Disease Surveillance System between January 2012 and December 2022, and Australian traveller movement data.

**Results:**

Between 2012 and 2022, 13 343 dengue cases were reported in Australia (median 1466 annual cases); 12 568 cases (94.2%) were imported, 584 (4.4%) were locally acquired and 191 (1.4%) had no origin recorded. Locally acquired cases decreased from a peak in 2013 (*n* = 236) to zero in 2021–22. Annual incidence of imported dengue ranged from 8.29/100 000 (*n* = 917 cases) to 22.10/100 000 (*n* = 2203) annual traveller movements between 2012 and 2019, decreased in 2020 (6.74/100 000 traveller movements; *n* = 191) and 2021 (3.32/100 000 traveller movements; *n* = 10) during COVID-19-related border closures, then rose to 34.79/100 000 traveller movements (*n* = 504) in 2022. Imported cases were primarily acquired in Southeast Asia (*n* = 9323; 74%), Southern and Central Asia (*n* = 1555; 12%) and Oceania (*n* = 1341; 11%). Indonesia (*n* = 5778; 46%) and Thailand (*n* = 1483; 12%) were top acquisition countries. DENV-2 (*n* = 2147; 42%) and DENV-1 (*n* = 1526; 30%) were predominant serotypes.

**Conclusion:**

Our analysis highlights Australia’s successful control of locally acquired dengue with *Wolbachia*. Imported dengue trends reflect both Australian travel destinations and patterns and local epidemiology in endemic countries.

## Introduction

Over 3.9 billion people across >128 countries are at risk for dengue.^1^ Global incidence has increased 30-fold over the past 50 years^2^ and an estimated 100–400 million infections occur annually, with 70% in the Asia-Pacific region.^1^^,^^3^ Dengue results from infection with any of four dengue virus serotypes (DENV-1 to DENV-4), which are transmitted to humans by *Aedes aegypti* and *Aedes albopictus* mosquitoes.^2^ While many infections are asymptomatic, severe disease may occur. There are no approved antivirals and treatment is supportive.^2^ Two dengue vaccines, Dengvaxia and Qdenga, are currently available with varying recommendations and safety implications.

Urbanization and climate change have expanded the geographic range of dengue virus and its mosquito vectors.^4^ Transmission dynamics vary, with year-round transmission, seasonal and periodic epidemics occurring in different settings.^1^^,^^4^ Dengue is the leading cause of febrile illness in returned travellers from all continents except Africa,^5^ and importation by travellers can drive outbreaks in non-endemic areas with competent vectors.^2^^,^^6^ Australia is not dengue endemic, but importation by viraemic travellers has led to local transmission and periodic outbreaks in areas of northern Queensland with competent vectors.^7–9^

Randomized and non-randomized field trials in multiple countries over the past decade have demonstrated the effectiveness of the *Wolbachia* replacement method in significantly reducing dengue transmission,^10–14^ when used as an adjunct to existing strategies including vector surveillance and control, mosquito avoidance, disease surveillance, case management, education and emergent vaccination programmes. Field releases of *Wolbachia-*infected *Ae. aegypti* as a method for controlling dengue were first conducted in two suburbs of Cairns, in northern Queensland in 2011,^7^^,^^15^ with the aim of stably introducing the maternally inherited intracellular bacterium *Wolbachia* into the local *Ae. aegypti* population and thereby reducing the mosquitoes’ ability to transmit dengue and other arboviruses.^16^ Phased deployment of *Wolbachia* mosquitoes throughout areas of northern Queensland between 2011 and 2019 has significantly reduced local dengue transmission, with sustained high levels of *Wolbachia* mosquitoes and the effective elimination of autochthonous dengue transmission as a public health concern.^7^^,^^15^^,^^17^

In the Australian context, analysis of dengue surveillance data enables both an evaluation of imported dengue trends and the impact of public health control initiatives in areas with local dengue transmission. We report on the epidemiological trends in imported and locally transmitted dengue in Australia from January 2012 to December 2022, and contextualize our findings with broader dengue epidemiological trends in the Asia-Pacific region and *Wolbachia* mosquito releases in northern Queensland.

## Methods

### Data sources

#### Dengue cases in Australia

We collated de-identified data on all laboratory-confirmed and clinically diagnosed dengue cases notified to the National Notifiable Disease Surveillance System (NNDSS) between 1 January 2012 and 31 December 2022. Data were provided by the Australian Government’s Office of Health Protection in July 2023. Details of variables obtained are in Table S1.

### *Wolbachia* implementation

We gathered data on *Wolbachia* mosquito release timelines in northern Queensland from published sources^15^^,^^17^ and operational data provided by the World Mosquito Program.

#### Traveller movement estimates

We sourced Australian traveller movement data from 2012 to 2022 from the Australian Bureau of Statistics (ABS) (Table S1, available at *JTM* online). Traveller movements are defined not by individual travellers or trips but rather by international border crossings categorized as short-term (<12 months overseas) resident departures (STRD; 2012–17) and returns (STRR; 2017–22).

Countries were grouped into geographical regions according to the Standard Australian Classification of Countries 2016 (Table S1, available at *JTM* online).

#### Dengue cases in other countries of interest

Where available, we collated publicly accessible data on dengue cases notified to national Departments of Health, other relevant national agencies or to the World Health Organisation for the most common countries of dengue acquisition (Table S2, available at *JTM* online). We searched PubMed, Google Scholar and Google databases to identify academic papers and reports documenting dengue epidemiology and/or burden of disease data, which were used when other surveillance data were unavailable.

### Epidemiological analysis

We estimated the number and annual incidence rate of imported dengue cases in Australia nationally and by jurisdiction. Region-specific incidences were calculated for the top three dengue acquisition regions and country-specific incidences for the top 10 countries of acquisition. We calculated incidence rates for imported cases by annual traveller movements using the number of imported cases as the numerator and ABS traveller movement data as the denominator. We calculated the annual local incidence of dengue for the top acquisition countries using national case numbers as the numerator and population data for each country as the denominator (Table S1, available at *JTM* online).

The project was approved by the Monash University Human Research Ethics Committee (project #28955) and Communicable Disease Network Australia jurisdictional members. Data were analysed using STATA version 15 (StataCorp. 2017. *Stata Statistical Software: Release 15*. College Station, TX: StataCorp LLC).

## Results

Between 1 January 2012 and 31 December 2022, a total of 13 343 dengue cases were notified to the NNDSS (median 1466 cases; range 11–2238). Of these, 12 568 cases (94%) were imported, 584 (4%) were locally acquired and 191 (1%) had no origin recorded.

### Locally acquired dengue cases

**Figure 1:**
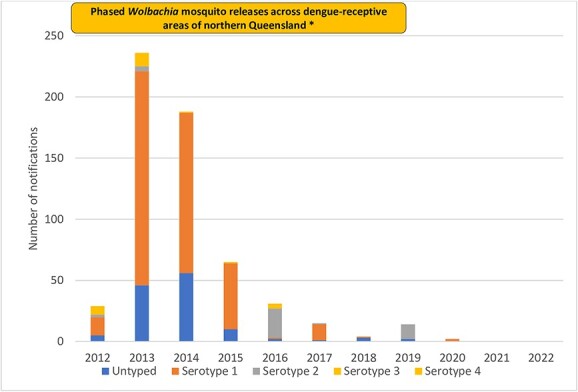
Number of locally acquired dengue cases and dengue serotype, Australia 2012–22. *Pilot releases of *Wolbachia* mosquitoes in two suburbs of Cairns occurred in 2011, followed by phased releases across urban areas of northern Queensland in 2013–2019^15^^,^^17^

Locally acquired cases decreased over time from a peak of 236 cases in 2013 to zero cases in 2021 and 2022 (Figure 1). Queensland (QLD) accounted for the vast majority of locally acquired cases (*n* = 560; 96%) (Table 1). Males accounted for 312 cases (53%) and 210 cases (36%) were in adults aged 40–59 years (Table 1). Dengue virus serotype data were available for 78% (*n* = 459) of cases, with DENV-1 predominating (*n* = 392, 67%), except in 2016 and 2019, when DENV-2 was more frequent (Figure 1).

**Table 1:** Epidemiological characteristics of notified dengue cases, Australia 2012–2022

Characteristics	Locally acquired dengue cases *N* = 584 (%)	Imported dengue cases *N* = 12 568 (%)	Cases with no origin recorded *N* = 191 (%)
*Sex* Male Female NI	312 (53) 272 (47)	6437 (51) 6107 (49) 26 (<1)	101 (53) 88 (46) 2 (1)
*Age* < 5 years 5–19 years 20–39 years 40–59 years ≥60 years	5 (<1) 79 (14) 198 (34) 210 (36) 92 (16)	83 (<1) 1090 (9) 5243 (42) 4536 (36) 1616 (13)	1 (<1) 10 (5) 100 (52) 58 (30) 22 (12)
*Jurisdiction* ACT NSW NT QLD SA TAS VIC WA	0 (0) 5 (<1) 0 (0) 560 (96) 6 (1) 0 (0) 9 (2) 4 (<1)	201 (2) 3079 (24) 446 (3) 1941 (15) 587 (5) 142 (1) 2831 (23) 3341 (27)	18 (9) 16 (8) 0 (0) 9 (5) 0 (0) 1 (1) 144 (75) 3 (2)
*Dengue virus serotype* 1 2 3 4 Mixed NI	392 (67) 43 (7) 22 (4) 2 (<1) 0 (0) 125 (21)	1526 (12) 2147 (17) 997 (8) 419 (3) 54 (<1) 7425 (59)	13 (7) 22 (11) 10 (5) 5 (3) 8 (5) 133 (70)

*Abbreviations*: ACT: Australian Capital Territory; NSW: New South Wales; NT: Northern Territory; QLD: Queensland; SA: South Australia; TAS: Tasmania; VIC: Victoria; WA: Western Australia; NI: no information

### Imported dengue cases

**Figure 2:**
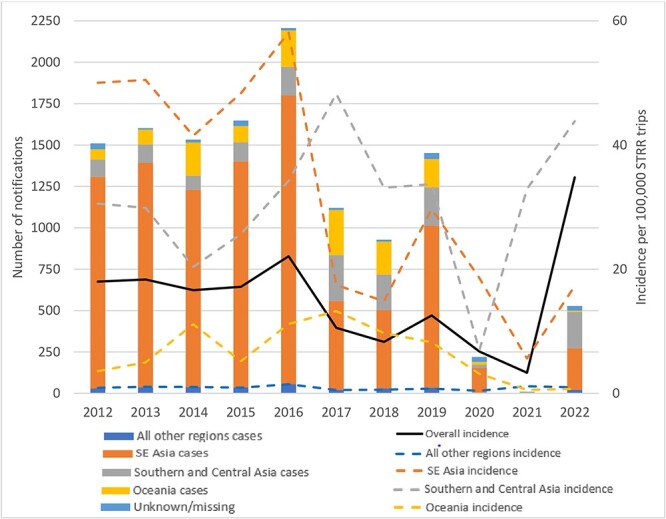
Number and notification incidence of imported dengue cases, Australia 2012–22. Abbreviations: STRR: short-term resident return; SE: Southeast. Vertical bars depict the number of cases by region and use the left *y*-axis, while the lines depict the overall (solid line) and regional (dotted line) notification incidences per 100 000 STRR trips and use the right *y*-axis. All other regions include Northwest Europe, Southern and Eastern Europe, North Africa and the Middle East, Sub-Saharan Africa, Northeast Asia

Imported cases were primarily acquired in SE Asia (*n* = 9323; 74%), followed by Southern and Central Asia (*n* = 1555; 12%) and Oceania (*n* = 1341; 11%) (Table S3, Figure S1a, available at *JTM* online). Young adults aged 20–39 years were the most frequently affected (*n* = 5243; 42%), with males comprising 51% (*n* = 6437) of cases (Table 1). Between 2012 and 2019, the estimated incidence of imported dengue notifications ranged from 8.29/100 000 (*n* = 917 cases) to 22.10/100 000 (*n* = 2203) annual traveller movements, with a peak in 2016 (Figure 2). Notification incidence decreased in 2020 [6.74/100 000 traveller movements (*n* = 191 cases)] and 2021 [3.32/100 000 traveller movements (*n* = 10)], coinciding with COVID-19-related international border closures and travel restrictions. In 2022, notification incidence rebounded to 34.79/100 000 (*n* = 504) traveller movements (Figure 2). By jurisdiction, Western Australia reported the most cases (*n* = 3341; 27%, Table 1), but notification incidence was greatest in the Northern Territory (NT) (mean 51/100 000 traveller movements) (Figure S1b, available at *JTM* online). From 2012 to 2016, the highest notification incidence by region was observed in travellers to Southeast (SE) Asia, whereas from 2017 onwards it was greatest for travellers to Southern and Central Asia (Figure 2). Imported dengue case notifications from all regions were low in 2020 and 2021, but a rebound was seen in 2022, particularly in travellers to Southern and Central Asia (Figure 2; Table S3, available at *JTM* online).

**Figure 3:**
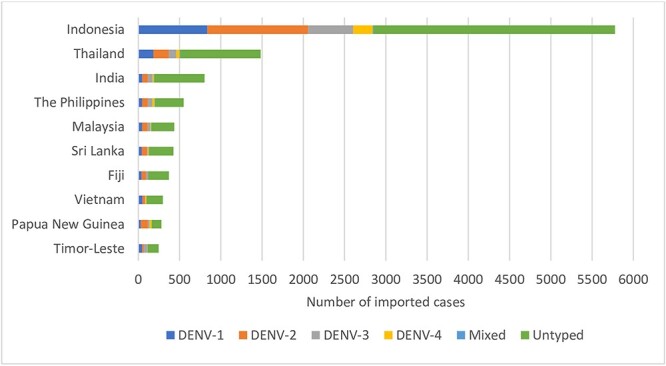
Top 10 countries of acquisition by DENV serotype for imported dengue cases, 2012–22. *Abbreviations*: DENV: Dengue virus. Ranking is based on number of cases imported from each country

**Figure 4:**
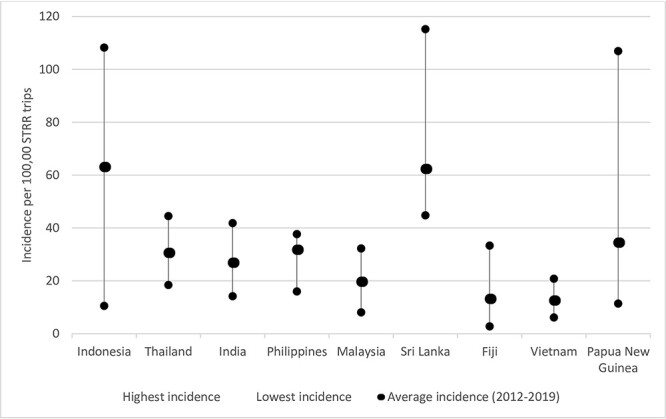
Lowest, highest and average incidence (per 100 000 traveller movements) for top countries of acquisition, 2012–19. *Abbreviations*: STRR: short-term resident return. STRR trips are obtained for each country from ABS data. Data for Timor-Leste not available. Average data for 2012 to 2019 only included as expected decreases in incidence occurred in 2020 following implementation of travel restrictions due to the COVID-19 pandemic

The top 10 countries of dengue acquisition were Indonesia (46%; *n* = 5778, peak notification year 2016), Thailand (12%; *n* = 1483 cases, peak in 2012), India (6%; *n* = 803, peak in 2017), the Philippines (4%; *n* = 550, peak in 2019), Malaysia (3%; *n* = 435, peaks in 2014 and 2015), Sri Lanka (3%; *n* = 426, peak in 2017), Fiji (3%; *n* = 370, peak in 2019), Vietnam (2%; *n* = 297, peak in 2019), Papua New Guinea (PNG) (2%; *n* = 280, peak in 2016) and Timor-Leste (2%; *n* = 246, peak in 2012) (Figure 3; Figure S2, Table S3, available at *JTM* online). Figure 4 displays the average, lowest and highest annual incidences (per 100 000 tmy) for these countries from 2012 to 2019; Indonesia, Sri Lanka and PNG exhibited both the highest average incidence and widest range during this period.

Serotype data were available for 41% (*n* = 5143) of imported cases, with DENV-2 (*n* = 2147; 42%) and DENV-1 (*n* = 1526; 30%) the predominant serotypes (Table 1). The dominant serotype varied by region and year (Figures S3 and S4, available at *JTM* online). DENV-2 was the most common serotype from all regions except for the Americas (DENV-1) (Figure S4, available at *JTM* online), and in all years except 2013–14 (DENV-1), 2020 (DENV-1) and 2022 (DENV-3) (Figure S3, available at *JTM* online).

**Figure 5:**
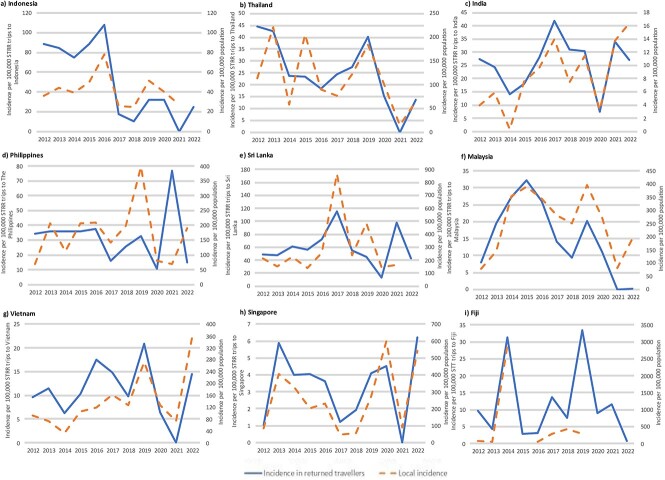
Estimated notification incidence of imported dengue in returned Australian travellers and estimated local incidence of dengue for countries of interest, 2012–22. *Abbreviations:* STRR: Short term resident return. The solid line represents the incidence in returned travellers and follows the left y-axis. The dashed line represents country specific local incidence and follows the right y-axis. Country-specific dengue case data derived from surveillance data for Indonesia, Thailand, India, Sri Lanka, Malaysia, Vietnam, The Philippines. Local incidence data for Fiji obtained from surveillance reports (Appendix, Table S1) and the literature.^53^ Data for local dengue cases not available for 2015 for Fiji. Data for local dengue cases not available for 2020 for Fiji. Data for local dengue cases not available for 2021 for Fiji. Data for local dengue cases not available for 2022 for Indonesia, Sri Lanka, Fiji. Data for local dengue cases for Vietnam available until 19th November 2020, 19th December 2021 and 12th December 2022. National dengue data not available for PNG or Timor-Leste. Traveller movement data not available for Timor-Leste. Singapore included given its status as the only other dengue endemic country in the top ten most frequented travel destinations (Appendix, Table S4).

Country-specific notification incidence rates for cases imported to Australia generally followed quite similar patterns to local dengue incidence, especially for Indonesia, Thailand, Malaysia, Vietnam, Singapore, India, Sri Lanka and Fiji (Figure 5).

## Discussion

Our study provides insights into trends in imported and locally acquired dengue in Australia over the last decade, a period marked by record dengue outbreaks in many countries, including in the Asia Pacific.^18^ Dengue is among the most frequently imported infections to Australia,^19^ and importation into areas of northern Queensland with competent mosquito vectors has historically led to secondary transmission and local outbreaks. We found that imported dengue notifications rose from 2012 to 2016, with peak notification years in 2016 and 2019 aligning with global trends,^18^ while locally acquired dengue cases declined to zero concurrent with the implementation of the *Wolbachia* method in northern Queensland. A sharp decline in imported cases from 2020 to 2021 corresponded with border closures and travel restrictions imposed due to the COVID-19 pandemic. Imported case notifications increased in 2022 following reopening of Australia’s borders, but locally acquired cases remained absent. Although DENV serotype distribution varied by region and year, our findings are consistent with a systematic review indicating DENV-2 and DENV-1 as the most common observed serotypes in outbreaks globally from 1990 to 2015.^20^

These broad trends are consistent with studies from other non-endemic settings. In the USA, imported cases increased from 2012 to 2016 and were high in 2019.^21^ In Europe, imported dengue case numbers remained relatively stable between 2015 and 2018 before a significant increase in 2019.^22^^,^^23^ However, Australia’s geographic location and the unique travel patterns and migration trends of Australians underpin some notable differences. We found that Asia and Oceania were the most common regions of acquisition, contrasting with the USA, where cases are primarily imported from the Americas.^24^^,^^25^ Similarly, we saw very few cases from Africa, which tends to contribute a higher proportion of imported cases to both the USA and Europe.^22^^,^^24^ These findings highlight the value of Australian dengue surveillance in providing sentinel information on circulating DENV serotypes and outbreaks in acquisition countries, especially in Asia and Oceania. These data can support surveillance programmes in countries with limited resources and surveillance capabilities and assist with risk assessment for travellers to these endemic regions.

Almost two-thirds (64%) of imported cases to Australia originated from Indonesia, Thailand and India, reflecting their popularity as travel destinations and aligning with previous research implicating them as common sources of imported dengue cases globally.^24^ Importation trends generally aligned with local dengue epidemiology, consistent with previous GeoSentinel reports indicating that dengue trends in returned travellers often mirror local outbreaks in destination countries.^26^^,^^27^ Anomalies in reported dengue incidence in Australia can thus serve as global alerts, signalling possible outbreaks to specific countries and the global health community.

Data regarding specific locations where Australians acquire dengue within Indonesia, where the *Wolbachia* method has been implemented in the Special Region of Yogyakarta in Java^11^ and further expansion is planned, are not available. However, Bali, which reports the highest annual dengue incidence in Indonesia^28^ and is a popular travel destination for Australians, is likely an important source of importations.^18^ Relatively low dengue incidence in Indonesia in 2017–18, potentially combined with a decline in tourism following the 2017 eruption of Bali’s Mt. Agung volcano,^29^ was reflected in lower imported dengue rates from Indonesia in 2017–18. India experienced large dengue outbreaks in 2021 and 2022, correlating with a marked increase in imported cases to Australia in 2022. This coincided with travel to India rebounding to 77% of 2019 levels. Almost 3% of the Australian population were born in India (Table S1, available at *JTM* online), making it a common travel destination, particularly for ‘visiting friends and relatives’ travellers.^30^ Trends seen for other common acquisition countries, such as the Philippines and Sri Lanka, likely reflect a combination of migration and travel trends, alongside a high local disease burden.^31^^,^^32^ Reasons for some inconsistencies in imported dengue incidence compared to local epidemiology (e.g. for Thailand in 2015) remain uncertain but might reflect spatial variability in local dengue incidence in destination countries.

Oceania (*n* = 1341 cases; 11%) was well represented as a source of dengue acquisition, with Fiji and PNG contributing almost half of all cases from this region. Interestingly, despite travel to Fiji returning to 92% of 2019 levels in 2022, only two imported cases were reported. The reasons for this are uncertain, but a possible contributing factor could be a true decrease in local incidence related to implementation of the *Wolbachia* method throughout Fiji’s three largest cities Suva, Nadi and Lautoka in 2018–19.^33^ Lack of robust national surveillance data from certain countries in Oceania highlights the value of Australian traveller data in discerning regional dengue trends.

Despite high numbers of imported dengue cases between 2012 and 2019, we observed a substantial reduction in locally acquired dengue cases in Australia over time. This almost certainly reflects the successful large-scale roll-out of *Wolbachia* mosquitoes in northern Queensland between 2013 and 2019. Long-term monitoring data indicate that *Wolbachia* has been self-sustaining at a high prevalence in local mosquito populations for a decade post-release.^34^ The most recent locally acquired dengue outbreak in Queensland occurred in Rockhampton in 2019 (13 laboratory-confirmed cases),^35^ an area where *Wolbachia* mosquitoes were not deployed. This marked the first locally acquired dengue outbreak in Central Queensland in 65 years, emphasizing the need for ongoing monitoring of areas in Australia with potential for local transmission. Given the absence of competent vectors outside QLD, the small number of locally acquired cases (*n* = 24) notified by other jurisdictions are potential misclassifications.

The success of the *Wolbachia* method in interrupting local dengue transmission in Australia is an encouraging development for other non-endemic settings with competent mosquito vectors like the USA and Europe, where sporadic outbreaks occur following importation.^21^^,^^22^^,^^36^ Globalization and climate change have led to geographic expansion of vectors,^4^ resulting in an increase in autochthonous dengue cases and outbreaks, including in areas not previously reporting local transmission.^37–41^ For example, the USA has seen a rise in locally acquired cases since 2020, including California’s first locally acquired case in October 2023,^42^ despite a decrease in imported cases due to COVID-19-related travel restrictions.^21^ Continuing geographic expansion of *Ae. aegypti* vectors and the dengue virus is predicted,^43^^,^^44^ presenting substantial challenges in prevention and control efforts. The World Mosquito Program^33^ and others^45^^,^^46^ have undertaken *Wolbachia* mosquito releases in at least 13 dengue-endemic countries and one non-endemic country (Australia) to date. Further scale-up of the *Wolbachia* method in Indonesia is underway, and the impact of this initiative on the incidence of dengue among Australian travellers to Indonesia in the years ahead will be of particular interest. The *Wolbachia* method also has important implications for control of other arboviral infections,^4^ as laboratory studies have demonstrated that *Wolbachia* can modulate replication of yellow fever, Zika and chikungunya viruses in *Ae. Aegypti* mosquitoes.^47^

Dengue vaccines hold promise for reducing the epidemiological and economic burden of dengue in endemic areas, especially when integrated into a multifaceted approach including surveillance and vector control. However, their use in travellers is unclear. At present, two live attenuated tetravalent dengue vaccines, Dengvaxia and Qdenga, are commercially available in a number of countries. For both vaccines, phase III efficacy trials were conducted among children in dengue-endemic settings and data are limited outside of this population.^48^ Safety concerns around the use of Dengvaxia limit its utility in dengue-naïve individuals, including most travellers, and it has never been commercially available in non-endemic countries.^49^ For Qdenga, vaccine efficacy differs by serostatus and infecting serotype, with lower efficacy observed in individuals seronegative for dengue at baseline, especially against DENV-3 or DENV-4.^50^ In September 2023, the WHO recommended Qdenga be considered in routine immunization programmes in countries with high dengue transmission intensity.^49^ The benefit in travellers is likely to be for long-term or frequent travel to high-risk destinations in dengue-experienced travellers.^49^ However, there are potential limitations for dengue-naïve travellers heading to areas where DENV-3 and DENV-4 circulate. While our dataset showed DENV-2 and DENV-1 were predominant among typed cases notified in Australia in 2012–22, DENV-3 and DENV-4 still accounted for a substantial proportion of cases, and DENV-3 has re-emerged in recent large outbreaks in Bangladesh^51^ and Brazil.^52^ DENV serotype data are limited in endemic country national surveillance reports, highlighting a need for enhanced serotype surveillance to guide risk assessment of travellers as well as dengue prevention and control efforts.

### Limitations

Limitations of our study include incomplete data on some variables such as region of acquisition and characterization of DENV serotypes. Data captured in the NNDSS are dynamic and subject to retrospective revisions; therefore, the presented data represent a point-in-time analysis of DENV case notifications and may vary from data reported in published NNDSS and jurisdictional reports covering the same period. Notably, other variables of interest such as visitor and immigrant status, pre-travel healthcare, purpose and duration of travel and clinical data are not systematically collected by the NNDSS. This dataset may underestimate the denominator for imported dengue cases as it does not include visitors or migrants entering Australia. In addition, due to the short incubation period of dengue, Australian travellers diagnosed with dengue while overseas might not have been captured in the reported surveillance data. Despite these constraints, our results are based on a large national dataset and provide the most complete record of dengue notification trends over the last decade in Australia. However, since dengue importation is influenced by traveller demographics and destination preference, which can be dynamic and unpredictable, the trends observed in this study may not be generalisable to future patterns. Nonetheless, they highlight the importance of considering country-specific dengue epidemiological trends when assessing travel-associated health risks for travellers.

## Conclusion

Our study highlights that notification trends of imported dengue in Australia reflect the travel destinations and patterns of Australian travellers, together with the local epidemiology in endemic countries. Six of the top 10 destinations for Australian travellers in 2022 are highly endemic for dengue, highlighting the importance of ensuring travellers are aware of the risk of dengue and the need to protect themselves from mosquito bites. These findings can aid travel health practitioners to undertake detailed risk assessments and provide accurate education and advice to Australian travellers.

Traditionally, public health measures for dengue prevention and control have encompassed prompt case identification, vector surveillance and control, and human behavioural measures sure as mosquito avoidance. More recently, vaccination programmes have been introduced, with endemic and non-endemic countries employing a mix of approaches depending on their local contexts. The implementation of the *Wolbachia* method in northern Queensland and other Asia-Pacific and Latin American countries over the past decade has proven its public health value for dengue control. Expanding its implementation in both endemic and non-endemic areas has the potential to achieve sustained control of dengue and other *Aedes*-borne viruses, benefiting not only local communities but also travellers to these areas.

## Funding

This work was supported by the National Health and Research Council (NHMRC) Postgraduate Scholarship (grant number 2002792 to AS); NHMRC Fellowship (grant number APP1155005 to KL); NHMRC Investigator Grants (grant number 2017229 to SLM) and the Wellcome Trust (grant number 224459/Z/21/Z to KA). The NHMRC was not involved in study design; in the collection, analysis and interpretation of data; in the writing of the report; and in the decision to submit the paper for publication.

## Author contributions

AS was involved in the conceptualization, data collection and curation, formal data analysis, data interpretation, investigation, methodology, validation, visualization and writing of the original draft, and review and editing of the manuscript. AS undertook the literature search and created the tables and figures in the manuscript. SLM was involved in the conceptualization, methodology, data interpretation, validation, visualization, and review and editing of the manuscript as well as provision of resources to carry out the project and supervision. KL was involved in the conceptualization, methodology, data interpretation, validation, visualization, and review and editing of the manuscript as well as provision of resources to carry out the project and supervision. SLM and KL had direct access to the data and have verified the underlying data reported in the manuscript. SLM and KL contributed equally to the project and manuscript. KLA was involved in the data collection and interpretation and literature search of the *Wolbachia* method as well as review and editing of the manuscript.

## Author contributions

Asma Sohail (Conceptualization [Lead], Data curation [Lead], Formal analysis [Lead], Investigation [Lead], Methodology [Lead], Project administration [Lead], Validation [Lead], Visualization [Equal], Writing—original draft [Lead], Writing—review & editing [Equal]), Katherine Anders (Methodology [Supporting], Writing—review & editing [Supporting]), Sarah McGuinness (Conceptualization [Equal], Methodology [Supporting], Resources [Equal], Supervision [Equal], Validation [Supporting], Visualization [Supporting], Writing—review & editing [Equal]), and Karin Leder (Conceptualization [Equal], Methodology [Supporting], Resources [Equal], Supervision [Equal], Validation [Supporting], Visualization [Supporting], Writing—review & editing [Supporting])

##  

**Conflict of interest**: The authors do not declare any conflicts of interest.

## Data availability statement

Data collected for this study will not be made available to other parties.

## Supplementary Material

Dengue_supplementary_file_JTM_revision_clean_taae014

## Notes

Results from this study were presented at The Southern Cross Travel and Tropical Medicine Conference, 2023 held in Sydney, Australia 1–3 September 2023.
