# Supplementary material for: The epidemiology of imported and locally acquired dengue in Australia, 2012–2022
Source: J Travel Med. 2024 Jan 18;31(2):taae014. doi: 10.1093/jtm/taae014 (PMC10911064; doi:10.1093/jtm/taae014)
Supplement: Dengue_supplementary_file_JTM_revision_clean_taae014 [file dengue_supplementary_file_jtm_revision_clean_taae014.docx]

**Supplementary materials**

**Methods**

**Table S1: Data Sources**

| **Data source** | **Variables obtained** | **Time period** | **Date received/accessed** | **Comments** |
| --- | --- | --- | --- | --- |
| National Notifiable Disease Surveillance System*^1^ | Disease diagnosis, date of notification, state, age group, gender, country of acquisition, dengue virus serotype | 1^st^ January 2012 – 31^st^ December 2022 | 4^th^ July 2023 | Limited data for some variables available on the NNDSS dashboard |
| Australian Bureau of Statistics^2^ | Australian traveller movements^#^ | 1^st^ January 2012 – 31^st^ December 2022 | 30^th^ June 2023 | STRR traveller movement data were obtained to reflect short term holiday/business travel |
| Australian Bureau of Statistics^3^ | Country of birth of Australian residents | N/A | 1^st^ August 2023 |  |
| Australian Bureau of Statistics^4^ | Standard Australian Classification of Countries | N/A | 1^st^ August 2023 | Countries grouped into regions: Oceania and Antarctica, Northwest Europe, Southern and Eastern Europe, North Africa and the Middle East, Southeast Asia, Northeast Asia, Southern and Central Asia, Americas and Sub-Saharan Africa. Sudan and South Sudan are classified under North Africa and the Middle East. |
| United Nations Department of Economics and Social Affairs^5^ | Country populations | 2012 - 2022 | 5^th^ August 2023 | Data obtained for Indonesia, Thailand, India, Malaysia, The Philippines, Sri Lanka, Singapore, Vietnam, Fiji |

Abbreviations: NNDSS: National Notifiable Disease Surveillance System, STRR: short term resident return, N/A: not applicable

*The NNDSS is a passive surveillance system operational in Australia since 1991. Each Australian jurisdiction defines its own notifiable diseases list and receives data from doctors and/or laboratories. De-identified data for diseases on the National Notifiable Disease List (NNDL) are then forwarded onto the NNDSS. Enhanced surveillance by public health units includes a detailed travel history to identify the place of dengue acquisition. A case is classified as locally acquired if, during the exposure period (3-14 days prior to symptom onset), there is no travel outside Australia, but the individual has either resided in or visited regions within Queensland with competent mosquito vectors.

^#^Prior to July 2017, the Australian Bureau of Statistics recorded short term resident departures (STRD) rather than STRRs, and these data were used as a proxy for STRR from 2012 to July 2017.

**Table S2: Dengue cases in countries of interest**

Data on the number of locally reported cases were obtained from the following sources

| **Country** | **Title** | **Data source** | **Time period** | **Date of access** | **Available from** | **Comments** |
| --- | --- | --- | --- | --- | --- | --- |
| Indonesia | National notified dengue cases | OpenDengue^6^ (original source: Indonesia Ministry of Health) | 2012 – 2022 | 2^nd^ September 2023 | <https://opendengue.org/data> |  |
| Thailand | Disease Surveillance Report 506 | Bureau of Epidemiology, Department of Disease Control, Ministry of Public Health | 2012 - 2022 | 3^rd^ August 2023 | <https://www.moph.go.th> |  |
| India | Dengue Data Application: Dengue and Severe Dengue  Dengue/Dengue Haemorrhagic Fever Situation in India | World Health Organisation  National Centre for Vector-Borne Diseases Control, Directorate General of Health Services, Ministry of Health and Family Welfare | 2012 - 2017  2018 – 2022 | 3^rd^ August 2023  3^rd^ August 2023 | <https://www.who.int>  <https://www.mohfw.gov.in> | Data for 2012 – 2017 unavailable from the National Centre for Vector-Borne Diseases Control. |
| Sri Lanka | Dengue | National Dengue Control Unit, Ministry of Health | 2012 - 2022 | 3^rd^ August 2023 | https://[www.dengue.health.gov.lk/web/index.php/en/updates/disease](http://www.dengue.health.gov.lk/web/index.php/en/updates/disease) |  |
| Vietnam | National notified dengue cases  Dengue Situation Update Number 610  Dengue Situation Update Number 636  Dengue Situation Update Number 661 | OpenDengue^6^ (original source: WHO WPRO)  World Health Organisation  World Health Organisation  World Health Organisation | 2012 – 2019  2020  2021  2022 | 30^th^ September 2023  30^th^ September 2023  30^th^ September 2023  30^th^ September 2023 | <https://opendengue.org/data>  <https://iris.who.int/bitstream/handle/10665/330698/Dengue-20201217.pdf>  <https://iris.who.int/bitstream/handle/10665/341149/Dengue-20211229.pdf>  <https://iris.who.int/bitstream/handle/10665/352792/Dengue-20221215.pdf> | Data for 2020 – 2022 unavailable from OpenDengue.  2020 data available until 29^th^ November 2020; 2021 data available until 19^th^ December 2021; 2020 data available until 12^th^ December 2022. |
| The Philippines | National notified dengue cases | OpenDengue^6^ (original source: WHO WPRO) | 2012 – 2022 | 29th September 2023 | <https://opendengue.org/data> |  |
| Malaysia | National notified dengue cases | OpenDengue (original source: WHO WPRO) | 2012 – 2022 | 29^th^ September 2023 | <https://opendengue.org/data> |  |
| Singapore | Dengue Surveillance data | National Environment Agency | 2012 – 2022 | 3^rd^ August 2023 | <https://www.nea.gov.sg> |  |
| Fiji | Dengue Fever | Ministry of Health and Medical Services | 2012 | 3^rd^ August 2023 | <https://www.health.gov.fj/dengue-fever> |  |

Abbreviations: WHO: World Health Organisation; WPRO: Western Pacific Regional Office

**Results**

**Table S3: Imported dengue notifications by year for the most common regions of acquisition and the top three countries of acquisition for each region, 2012-2022**


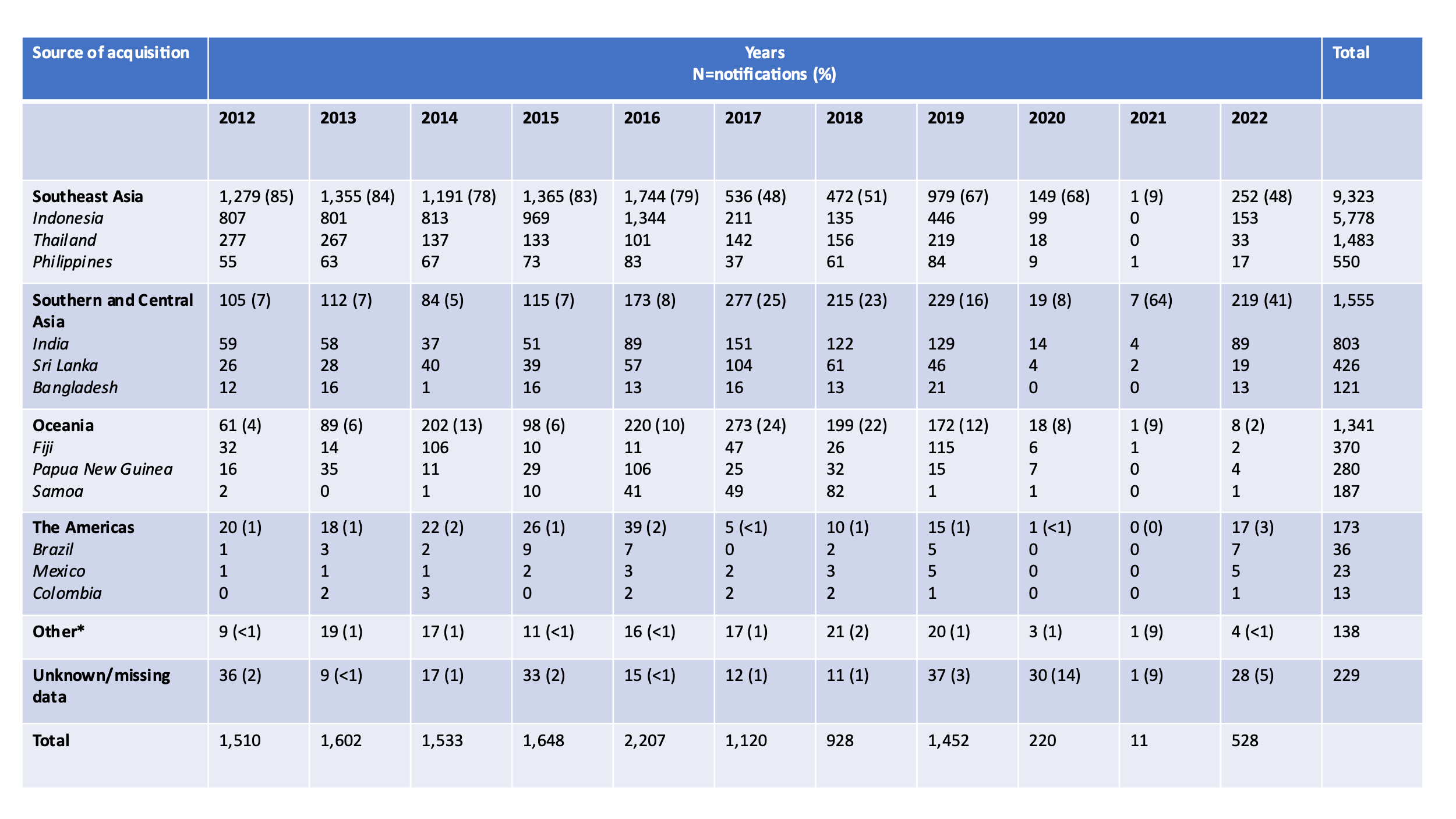


*Other includes Northeast Asia, North-western Europe, Southern and Eastern Europe, North Africa and the Middle East, Sub-Saharan Africa

The percentages are calculated as column percentages

**Figure S1a: Notifications of imported dengue cases by jurisdiction and region of acquisition, Australia, 2012-2022**

Abbreviations: ACT: Australian Capital Territory; NSW: New South Wales; NT: Northern Territory; QLD: Queensland; SA: South Australia; TAS: Tasmania; VIC: Victoria; WA: Western Australia; SE: Southeast

**Figure S1b: Notification incidence of imported dengue cases by jurisdiction, Australia, 2012-2022**

Abbreviations: STRR: Short term resident return; ACT: Australian Capital Territory; NSW: New South Wales; NT: Northern Territory; QLD: Queensland; SA: South Australia; TAS: Tasmania; VIC: Victoria; WA: Western Australia

*The number of traveller movements for each jurisdiction were calculated according to the jurisdiction that travellers indicated as ‘state of residence’ on the incoming passenger card completed by all international arrivals on entry into Australia

**Figure S2: Number of notifications and notification incidence of imported dengue cases for top two countries of acquisition from Southeast Asia, Southern and Central Asia and Oceania, 2012-2022**


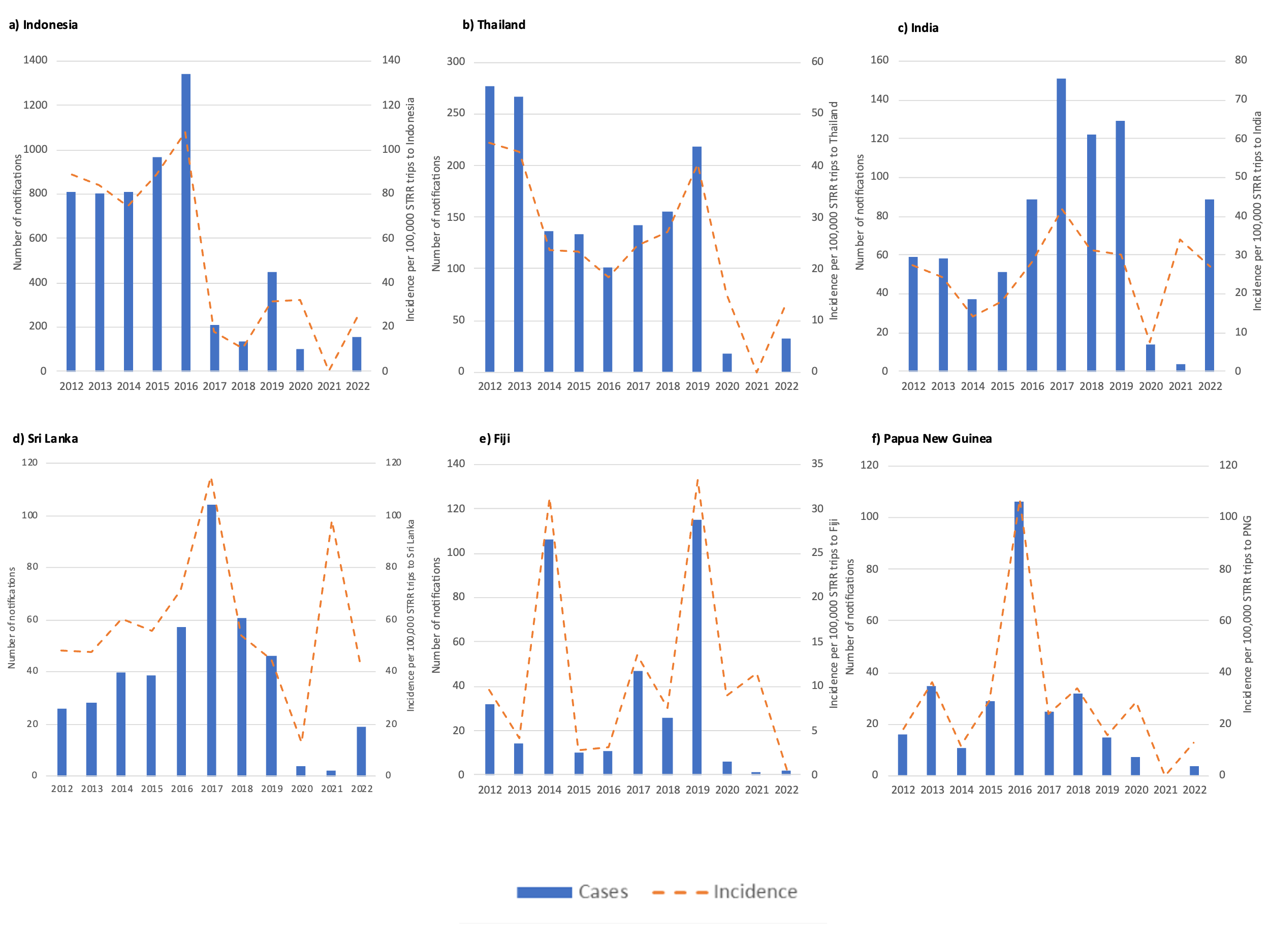


Abbreviations: STRR; short term resident return

Vertical bars depict the number of cases and use the left y-axis, while the line depicts the notification incidence per 100,000 STRR trips and uses the right y-axis

**Figure S3: Dengue virus serotype of imported dengue cases, Australia 2012-2022**

Excludes untyped cases

**Figure S4: Dengue virus serotype for imported cases by region of acquisition, 2012-2022**


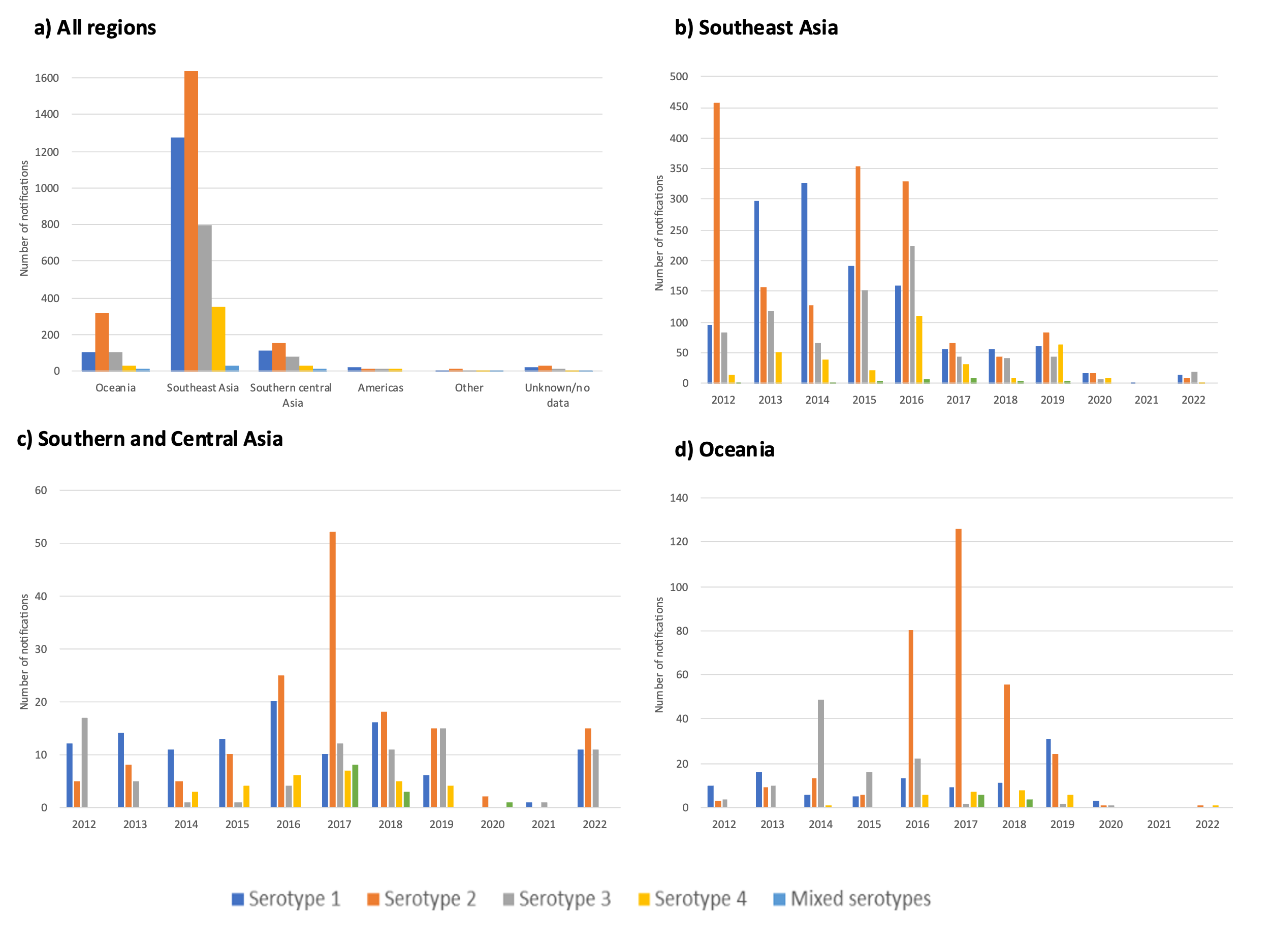


Excludes untyped cases

**Table S4: Top ten travel destinations for Australian travellers, 2012-2022**

**
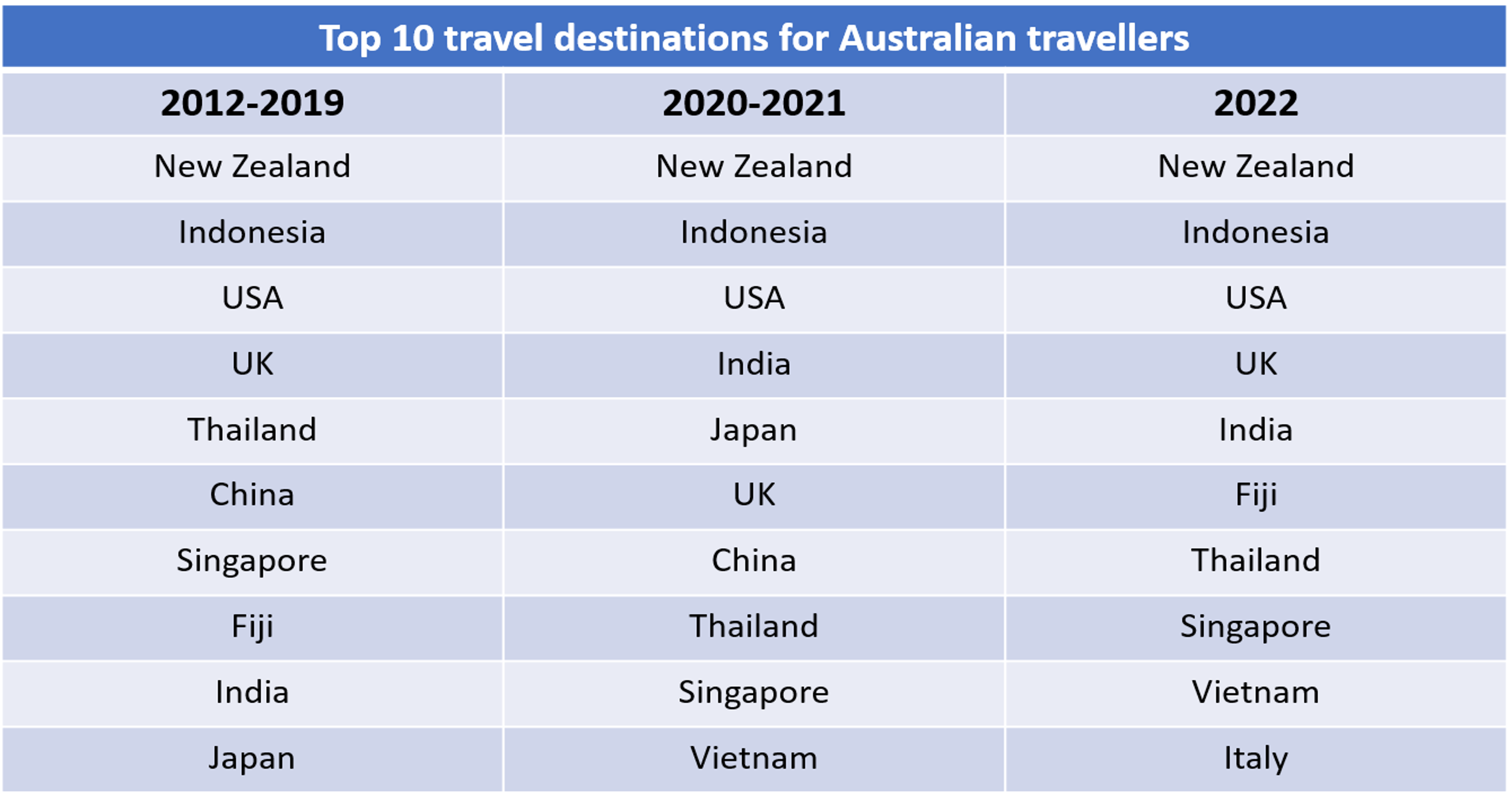
**

Abbreviations: USA: United States of America, UK; United Kingdom

Ranking is based on the number of short-term resident return trips

Three time periods have been chosen to delineate between pre-COVID-19 pandemic (2012-2019), during the COVID-19 pandemic when significant travel restrictions were in place (2020-2021) and post-COVID-19 pandemic (2022)

Australia’s international border re-opened in November 2021

**References:**

1. Australian Government Department of Health and Aged Care. National Notifiable Disease Surveillance System (NNDSS) Canberra, Australia: Australian Government Department of Health and Aged Care; 2023 [updated February 2023]. Available from: <https://www.health.gov.au/our-work/nndss#:~:text=The%20National%20Notifiable%20Diseases%20Surveillance,the%20impact%20of%20these%20diseases>.

2. Australian Bureau of Statistics. Overseas Arrivals and Departures, Australia Canberra, Australia: ABS; 2022. Available from: <https://www.abs.gov.au/statistics/industry/tourism-and-transport/overseas-arrivals-and-departures-australia/>.

3. Australian Bureau of Statistics. Migration, Australia Canberra, Australia: Australian Bureau of Statistics; 2021. Available from: <https://www.abs.gov.au/statistics/people/population/migration-australia/latest-release>.

4. Australian Bureau of Statistics. Standard Australian Classification of Countries (SACC): ABS; 2016. Available from: <https://www.abs.gov.au/statistics/classifications/standard-australian-classification-countries-sacc/latest-release>.

5. United Nations Department of Economics and Social Affairs. Data portal: Population Division: United Nations; 2023. Available from: <https://population.un.org/wpp/>.

6. Clarke J LA, Gupte PR, Pigott DM, van Panhuis WG, Wilbert G et al. OpenDengue: data from the OpenDengue database. Version [1.2] 2023. Available from: <https://doi.org/10.6084/m9.figshare.24259573.v3>.
